# Dataset of RGB images of healthy grapevine leaves and with downy mildew, powdery mildew, Esca complex, and erineum mite symptoms

**DOI:** 10.1016/j.dib.2026.112743

**Published:** 2026-04-02

**Authors:** Fernando Portela, Gabriel Carneiro, Leilson Ferreira, Cláudio A. Paredes, Joaquim J. Sousa, Emanuel Peres, Raul Morais, Luís Pádua

**Affiliations:** a Centre for the Research and Technology of Agro-Environmental and Biological Sciences (CITAB), University of Trás-os-Montes e Alto Douro, 5000-801 Vila Real, Portugal; b Agronomy Department, School of Agrarian and Veterinary Sciences, University of Trás-os-Montes e Alto Douro, 5000-801 Vila Real, Portugal; c Engineering Department, School of Science and Technology, University of Trás-os-Montes e Alto Douro, 5000-801 Vila Real, Portugal; d proMetheus, Research Unit in Materials, Energy and Environment for Sustainability, Escola Superior Agrária, Instituto Politécnico de Viana do Castelo, 4900-347 Viana do Castelo, Portugal; e Centre for Robotics in Industry and Intelligent Systems (CRIIS), INESC Technology and Science (INESC-TEC), 4200-465 Porto, Portugal; f CISAS—Center for Research in Agrifood Systems and Sustainability, Instituto Politécnico de Viana do Castelo, 4900-347 Viana do Castelo, Portugal; g Institute for Innovation, Capacity Building and Sustainability of Agri-Food Production (Inov4Agro), University of Trás-os-Montes e Alto Douro, 5000-801 Vila Real, Portugal

**Keywords:** Precision viticulture, *Vitis vinifera*, Diseases, Disease classification, Grapevine monitoring

## Abstract

This dataset consists of a collection of high-resolution RGB images of grapevine leaves, designed to support research in plant pathology, precision viticulture, and computer vision. The images were collected in situ from experimental and commercial vineyards in the north of Portugal, covering different vineyard conditions and management practices. The dataset includes healthy leaves from three grapevine Portuguese cultivars Loureiro, Viosinho and Malvasia Fina, photographed under natural lighting conditions without artificial adjustments. It is organized into five categories: healthy leaves and leaves showing symptoms of downy mildew (*Plasmopara viticola*), powdery mildew (*Erysiphe necator*), Esca complex and Erineum Mite (*Colomerus vitis*). Images are provided in JPEG format with a resolution of 3000 × 3000 pixels and 1024 × 1024 pixels and arranged in folders by health status and disease type. This dataset can be used for machine learning and deep learning applications in disease detection/classification, cultivar identification, and can support other precision agriculture applications, as well as being used for agricultural robotics and educational purposes. An evaluation on three deep learning architectures demonstrated the suitability of the dataset into separating the five classes.

Specifications Table

**Table:** 

Subject	Earth & Environmental Sciences
Specific subject area	RGB image dataset of grapevine leaves for disease detection and cultivar identification
Type of data	Images (JPG format)
Data collection	Images were collected from grapevine cultivars in natural vineyard environments. Leaves were photographed in situ, including healthy samples and those showing symptoms of downy mildew, powdery mildew, Esca complex, and erineum mite.
Data source location	• University of Trás-os-Montes e Alto Douro (41°17′13″N 7°44′08″W; 462 m), Folhadela, Vila Real, North of Portugal. • Casa de Mateus Foundation (41°17′40″N 7°42′53″W; 475 m), Mateus, Vila Real, North of Portugal. • Higher School of Agriculture of the Polytechnic Institute of Viana do Castelo (41°47'34"N 8°32'20"W; 50 m), Refóios do Lima, Ponte de Lima, North of Portugal. • Vineyards in Abaças (41°13′35.7″N 7°41′56.6″W; 566 m), Abaças, Vila Real, North of Portugal.
Data accessibility	Repository name: Zenodo Data identification number: https://doi.org/10.5281/zenodo.17343473 Direct URL to data: https://zenodo.org/records/17343473
Related research article	None

## 1 Value of the Data

• This dataset contains a collection of RGB images of grapevine leaves, collected under natural vineyard conditions, including both healthy samples and leaves manifesting symptoms of *Plasmopara viticola* (downy mildew), *Erysiphe necator* (powdery mildew), Esca complex, and erineum mite (Colomerus vitis). These diseases are among the most economically impactful in viticulture, and their symptoms can be visually subtle to detect and can vary under field conditions.
• The images were captured in real vineyard environments and in a non-destructive way, reflecting the natural variations in lighting, leaf orientation, and background. This makes the dataset suitable for developing and testing machine learning and deep learning models for disease classification and severity assessment.
• The dataset includes samples of healthy leaves from three Portuguese grapevine cultivars: Loureiro, Viosinho, and Malvasia Fina. The healthy leaf images can support cultivar identification, contributing to studies in ampelography, genotype-phenotype correlation, and automated classification of grapevine varieties based on leaf morphology.
• Researchers in the domains of plant phenotyping, agricultural robotics can use this dataset to design and evaluate algorithms and models for automated disease monitoring, that can help decision support systems in vineyard management for tasks such as spraying and in the implementation of other containment measures.
• The dataset can also be used for educational purposes, serving as a visual reference for students, agronomists and vineyard managers, as it provides examples of leaf symptoms from common grapevine diseases and cultivar-specific leaf characteristics.

## 2 Background

In precision viticulture, image-based approaches have become increasingly relevant for monitoring grapevine health and detecting early signs of disease. Technologies such as RGB imaging [[1], [2], [3]], multispectral cameras [4,5], LiDAR [6], and thermal sensors [7,8] are widely employed to assess plant stress and disease symptoms. These tools provide spatial and temporal insights into grapevine condition, supporting sustainable vineyard management and enabling timely interventions.

The availability of publicly accessible datasets has significantly contributed to the development of automated disease detection systems in viticulture [9]. For instance, datasets focusing on downy mildew symptoms in Merlot [10], Esca disease [11], and multispectral imagery for *flavescence dorée* [4], have advanced machine learning and computer vision applications. These resources have laid the foundation for robust classification models and decision-support tools, fostering precision agriculture practices.

Most RGB datasets currently available for grapevine disease detection have been acquired under controlled lighting and environmental conditions [10], often using isolated leaves captured against uniform or simplified backgrounds in laboratory or semi-controlled acquisition settings [12,13]. While these datasets have proven valuable for algorithm development, they may not fully capture the variability and complexity of real-world vineyard environments. Factors such as natural illumination, canopy architecture, and background interference introduce challenges that require more diverse and representative datasets to improve model generalization and reliability in operational contexts.

## 3 Data Description

**Fig. 1:**
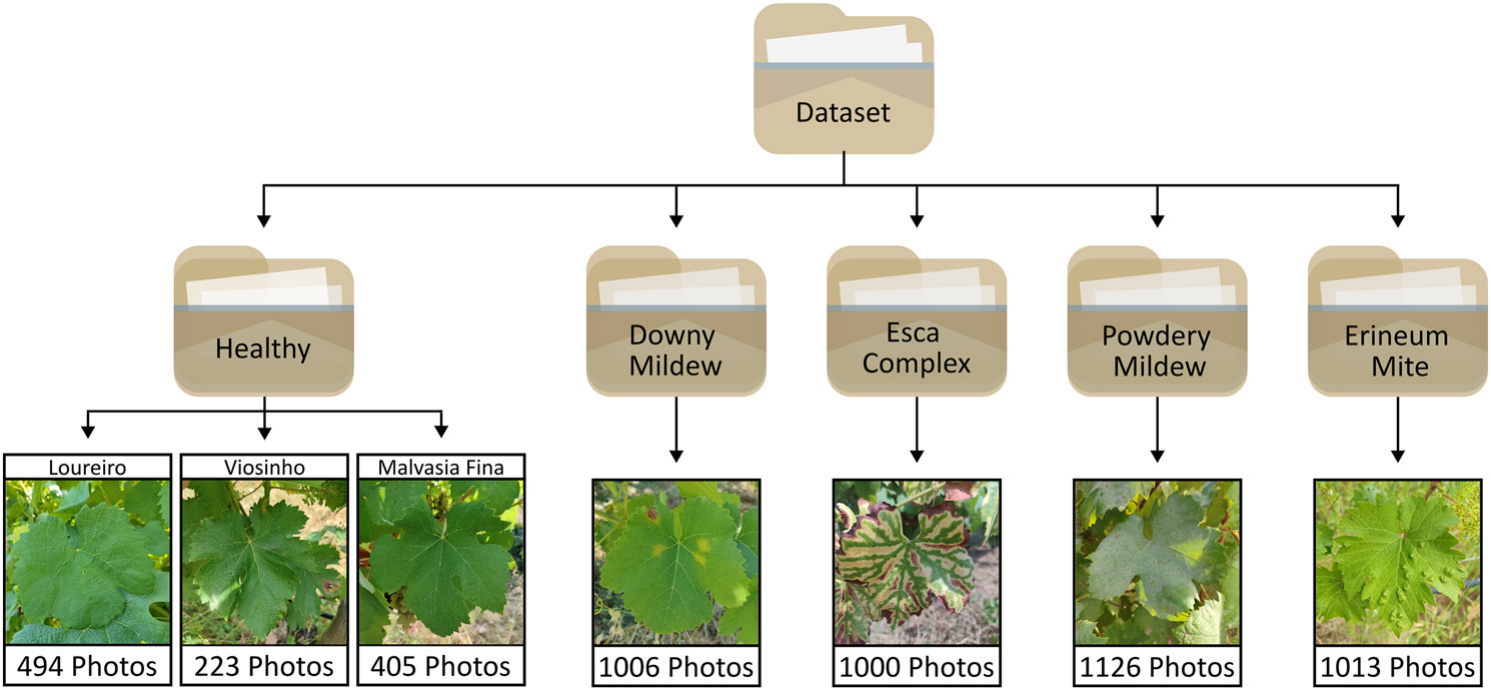
Dataset structure and representative examples of grapevine leaf categories, along with the total number of images.

The grapevine leaf image dataset is organized into a structured repository containing RGB images captured under natural field conditions. It is divided into five main folders: Healthy (494 images of Loureiro, 223 images of Viosinho, 405 images Malvasia Fina, totalling 1122 images), Downy Mildew (1006 images), Powdery Mildew (1126 images), Esca Complex (1000 images), and Erineum Mite (1013 images). Each folder corresponding to a specific class (Fig. 1). The dataset includes both healthy and diseased leaves from Portuguese grapevine cultivars. Healthy leaf images are useful for cultivar identification and differentiation from symptomatic leaves, while diseased leaf images are suitable for automated disease classification.

All images are stored in .JPEG format with a standardized resolution of 3000 × 3000 pixels and at a reduced size of 1024 × 1024. The acquisition was performed using a high-resolution smartphone RGB camera during daytime in vineyards during throughout the 2024 and 2025 growing seasons.

Each folder represents a specific disease class or the healthy status of grapevine leaves. The healthy folder includes images renamed with cultivar names, enabling the direct identification of the grapevine varieties. Therefore, filenames contain the acquisition timestamp followed by the cultivar name to support studies related to leaf morphology (e.g., YYYYMMDD_HHMMSS_variety). These images were collected in plots where cultivar identity was known for each plant. For symptomatic leaves, filenames include only the timestamp has many images were obtained in plots with more than one variety, where cultivar identification at the single‑leaf level was not reliable. All images are systematically organized to reflect their category and acquisition context (Table 1).

**Table 1:** Overview of grapevine foliar conditions in healthy leaves and leaves affected by diseases a description of visual characteristics and a representative photographic example.

Condition	Description	Visualization
1. Healthy	Uniformly green leaf with a smooth texture, no spots or deformities, minimal or no discolouration.	
2. Downy Mildew	Yellowish or light green blotches on the adaxial surface, irregular in shape; white to greyish mold on the underside under humid conditions. Spots turn brown and necrotic over time.	
3. Esca Complex	“Tiger stripe” pattern with alternating green and yellow-brown necrosis; black streaks may appear along veins; advanced stages cause leaf drying.	
4. Powdery Mildew	White, powdery fungal growth on both sides; leaves may curl upwards and develop necrosis in severe cases.	
5. Erineum Mite	Small blister-like swellings on the adaxial surface; affected areas may turn brown and dry over time, causing leaf deformation and reduced photosynthetic capacity.	

## 4 Experimental Design, Materials and Methods

### 4.1 Experimental design

**Fig. 2:**
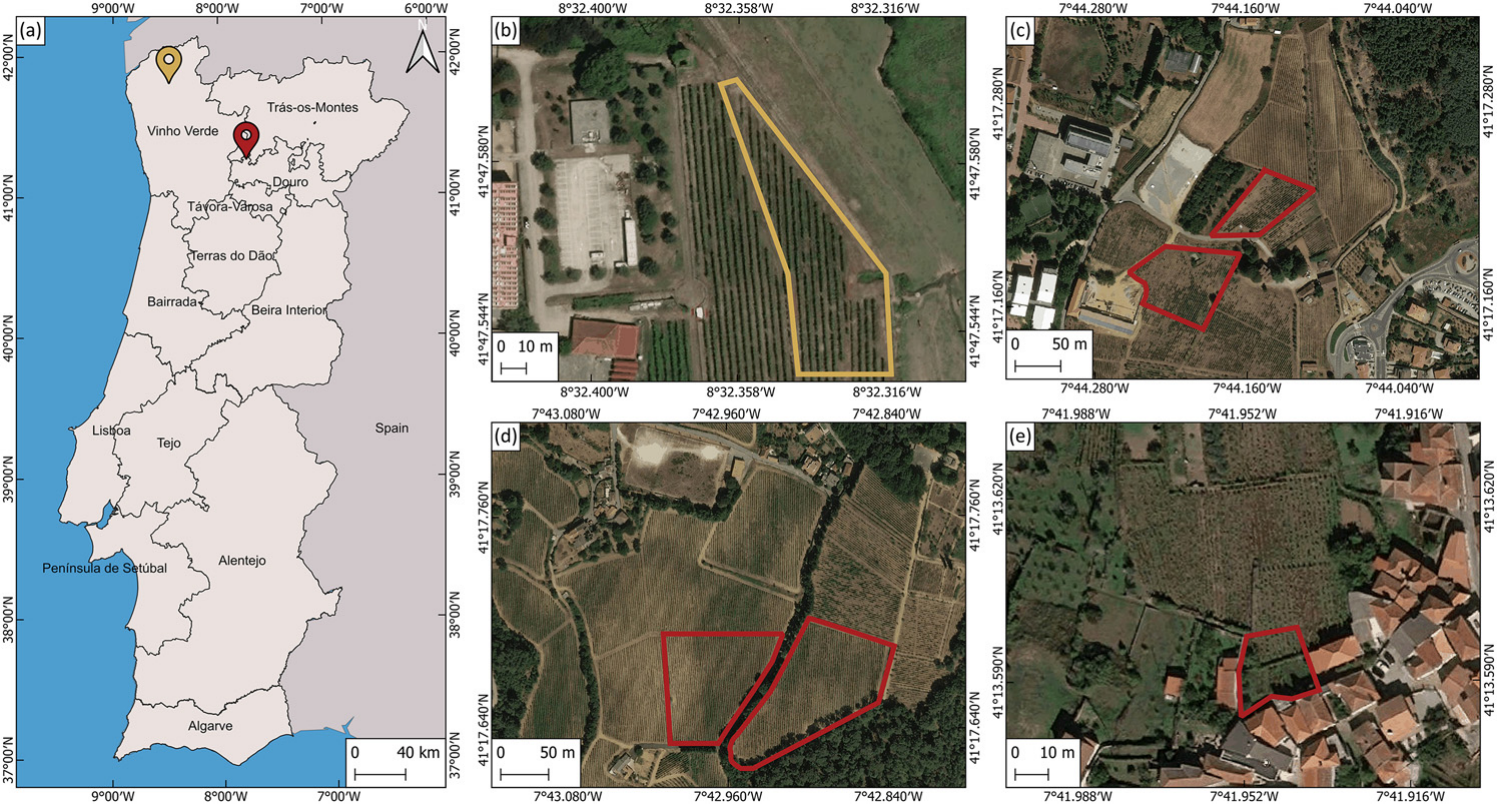
Location of the vineyards where images were collected: (a) general view of the location of the areas within mainland Portugal divided by wine regions, Vinho Verde region (yellow) and Douro region (red); (b) experimental vineyard plot of the Polytechnic Institute of Viana do Castelo; (c) experimental vineyard plots from the University of Trás-os-Montes e Alto Douro, (d) commercial vineyards from Casa de Mateus Foundation, and (e) small-scale vineyard in Abaças. Base map OrtoSat2023 true color image obtained from the National Geographic Information System (SNIG, Portuguese Directorate-General for Territory, Lisbon, Portugal).

The dataset was created through field-based image acquisition of grapevine leaves showing visual symptoms of major grapevine diseases under natural conditions. Image collection was carried out in four vineyard locations across two regions in northern Portugal (Fig. 2a): experimental vineyard at the Higher School of Agriculture of the Polytechnic Institute of Viana do Castelo (Fig. 2b); experimental vineyard plots at the University of Trás-os-Montes e Alto Douro (Fig. 2c); commercial vineyards of Casa de Mateus Foundation (Fig. 2d); and a small-scale vineyard plot in Abaças, Vila Real (Fig. 2e). These sites represent different terroirs and grapevine management practices, contributing to the variability of the dataset.

### 4.2 Image acquisition and dataset organization

Images were captured using a Redmi Note 12 Pro (Xiaomi Inc., Beijing, China) smartphone, equipped with a rear main camera with a 108MP wide sensor (f/1.9, 1/1.52″, 0.7 µm native pixel size), employing 9-in-1 pixel binning for an effective 2.1 µm pixel size, and a 6-element lens. All images were taken using the default camera application in a 1:1 aspect ratio, in automatic mode to maintain consistent exposure and focus settings. AI scene optimization and beauty enhancement features were disabled to avoid software-based alterations to color, texture, or contrast.

The acquisition process involved walking through vineyard rows and visually inspecting the grapevines for disease symptoms of downy mildew (*Plasmopara viticola*), powdery mildew (*Erysiphe necator*), erineum mite (*Colomerus vitis*), or Esca complex (*Phaeomoniella clamydospora, Phaeoacremonium aleophiluma e Fomitiporia* spp.). Table 2 presents a summary of the image acquisition dates and the number of photographs per disease.

**Table 2:** Temporal distribution of image acquisition for each disease class, including the healthy leaves.

Date	Healthy	Downy Mildew	Esca Complex	Powdery Mildew	Erineum Mite
28/06/2024	100	99	—	—	—
12/07/2024	91	100	—	—	—
30/07/2024	100	100	—	—	—
12/08/2024	104	100	—	—	—
23/08/2024	99	100	—	—	—
03/06/2025	—	166	1	—	45
18/06/2025	223	123	2	—	15
27/06/2025	—	218	7	—	143
14/08/2025	191	—	318	36	—
27/08/2025	180	—	427	—	407
28/08/2025	—	—	5	—	—
12/09/2025	15	—	137	71	—
01/10/2025	—	—	—	102	—
02/10/2025	—	—	72	124	25
09/10/2025	—	—	14	167	94
14/10/2025	—	—	1	583	178
23/10/2025	19	—	16	43	106
**Total**	1122	1006	1000	1126	1013

When symptomatic leaves were identified, photographs were usually taken directly on the grapevine, preserving their natural arrangement. During acquisition, leaves were positioned or framed so that the lamina was fully visible in most cases, without occlusion by other leaves, shoots or bunches, documenting the symptoms clearly on the leaf surface. To minimize motion blur and framing inconsistencies, images were captured at a distance of approximately 20 to 40 cm. Healthy leaves were also documented under the same conditions to provide reference data for disease discrimination and cultivar identification. The images were stored at their native resolution in JPEG format (Table 3), retaining EXIF metadata (timestamp, exposure settings, and, when available, GPS coordinates).

**Table 3:** Vineyard plots, geographical coordinates (WGS 84), and the grapevine diseases identified at each location. ESA-IPVC: Higher School of Agriculture, Polytechnic Institute of Viana do Castelo; UTAD: University of Trás-os-Montes e Alto Douro; FCM: Casa de Mateus Foundation; H: Healthy; DM: Downy Mildew; EC: Esca Complex; PM: Powdery Mildew; EM: Erineum Mite.

Location	Grapevine varieties	Coordinates	Disease(s)
ESA-IPVC	Loureiro	41°47′34.2″N 8°32′20.5″W	H, DM
UTAD	Malvasia fina	41°17′12.9″N 7°44′08.5″W	H, DM, EC, PM, EM
	White varieties	41°17′10″N 7°44′12″W	DM, EM
FCM	Viosinho	41°17′39.8″N 7°42′53.4″W	H, PM, DM, EM
	Sauvignon blanc	41°17′39.7″N 7°42′58.1″W	DM, EM
Abaças	White varieties	41°13′35.7″N 7°41′56.6″W	PM

**Fig. 3:**
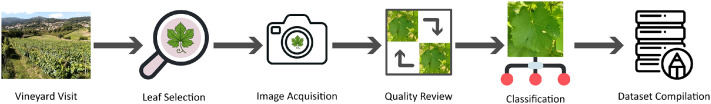
Workflow for dataset creation: vineyard visit, leaf selection, image acquisition, quality review, classification, and dataset compilation.

All images were taken under natural lighting and environmental conditions, without artificial manipulation (Fig. 3). This approach ensured realistic variability in leaf morphology, background elements, and illumination. After acquisition, images were manually reviewed, classified by disease type or health status, and organized into folders to facilitate the use in supervised learning workflows and image-based diagnostic systems. The image acquisition and the initial review and classification were performed by the same operator to ensure consistency in labeling. Subsequently, final quality control and verification of the annotations were conducted independently by a second reviewer. The dataset includes leaves from several grapevine cultivars (Table 3), with emphasis on Portuguese varieties such as Loureiro and Malvasia Fina.

### 4.3 Dataset evaluation using deep learning

Three state-of-the-art deep learning models were employed using the PyTorch framework: ConvNeXtV2 [14], Swin Transformer (Tiny) [15], and Vision Transformer (ViT-S) [16,17]. These architectures were selected for their performance in image classification and their different approaches to features representation. Each model contains approximately 30 million parameters, representing a comparable complexity and a balanced evaluation across different design paradigms. ConvNeXtV2 is effective on texture-rich images and performs well when training data is limited. Swin Transformer models both local and global image dependencies, which is useful for detecting symptoms that vary in scale or position across the leaf surface. ViT-S captures long-range contextual relationships, enabling the detection of dispersed or irregular disease patterns. The evaluation of these models provides a comparison of how modern architectures perform on grapevine disease classification tasks using the dataset. The models were not included as benchmark architectural performance, but to verify that the dataset supports accurate learning across modern architectures with different internal mechanisms (convolutional, hierarchical transformer, and pure transformer designs).

### 4.4 Data preparation and augmentation

The dataset was split into train (70%), validation (15%), and test (15%) subsets. All images were resized to 224 × 224 pixels before model input. Data augmentation during training included random cropping and rescaling, colour jittering (brightness, contrast, saturation, and hue), grayscale conversion with probability of 0.2, Gaussian blur (radius between 0.1 and 2.0), and horizontal flipping. All images were normalized using the ImageNet mean and standard deviation values [18]. For validation and testing, only resizing, centre cropping, and normalization were applied.

### 4.5 Model training

Each model was initialized with pre-trained weights and adapted for the classification task by replacing the original classification layer with a new block consisting of: a dropout layer (p = 0.2); a dense layer with 2048 units activated by the SiLU [19] function; and an output layer matching the number of classes.

Training was conducted in two stages. In the first stage, the pre-trained backbone was frozen, and only the new classification head was trained. In the second stage, all layers were unfrozen and fine-tuned with a reduced learning rate. Models were trained for 100 epochs with a batch size of 64, using the AdamW optimizer [20] and a cosine annealing learning rate schedule. Cross-entropy loss was used as the objective function, and performance was monitored using the macro-averaged F1-score. The model achieving the highest validation F1-score was selected for final testing. Confusion matrices and classification reports were generated to analyse class-level performance, and all the settings and metrics were documented.

### 4.6 Training performance

The dataset quality and consistency of the dataset were assessed through model performance metrics as presented in Table 4. All three architectures achieved high accuracy and balanced class-wise F1-scores. Among the evaluated models, ConvNeXtV2 obtained the best overall results, with an accuracy and macro-averaged F1-score of 0.97, followed by Swin Transformer (Tiny) and ViT-S. These results confirm the dataset enables both convolution-based and transformer-based models to achieve a near-perfect discrimination among its five classes.

**Table 4:** Performance metrics on the test set of the three evaluated models (ConvNextV2, Swin-T, and ViT-S) across the five classes. DM: Downy Mildew; EM: Erineum Mite; EC: Esca Complex; PM: Powdery Mildew. Best performances per metric highlighted in bold.

Class	ConvNextV2			Swin-T			ViT-S		
	*Precision*	*Recall*	*F1-Score*	*Precision*	*Recall*	*F1-Score*	*Precision*	*Recall*	*F1-Score*
DM	0.9737	**0.9801**	**0.9769**	0.9610	**0.9801**	0.9705	**0.9928**	0.9139	0.9517
EM	**0.9732**	0.9539	0.9635	0.9551	**0.9803**	**0.9675**	0.9359	0.9605	0.9481
EC	**0.9933**	**0.9933**	**0.9933**	**0.9933**	**0.9933**	**0.9933**	**0.9933**	**0.9933**	**0.9933**
Healthy	**0.9422**	**0.9702**	**0.9560**	0.9412	0.9524	0.9467	0.9314	**0.9702**	0.9504
PM	0.9759	0.9586	0.9672	**0.9875**	0.9349	0.9605	0.9706	**0.9763**	**0.9735**
*Accuracy*	**0.9709**			0.9671			0.9633		
Macro Average	**0.9711**	**0.9709**	**0.9709**	0.9675	0.9671	0.9671	0.9642	0.9633	0.9633

**Fig. 4:**
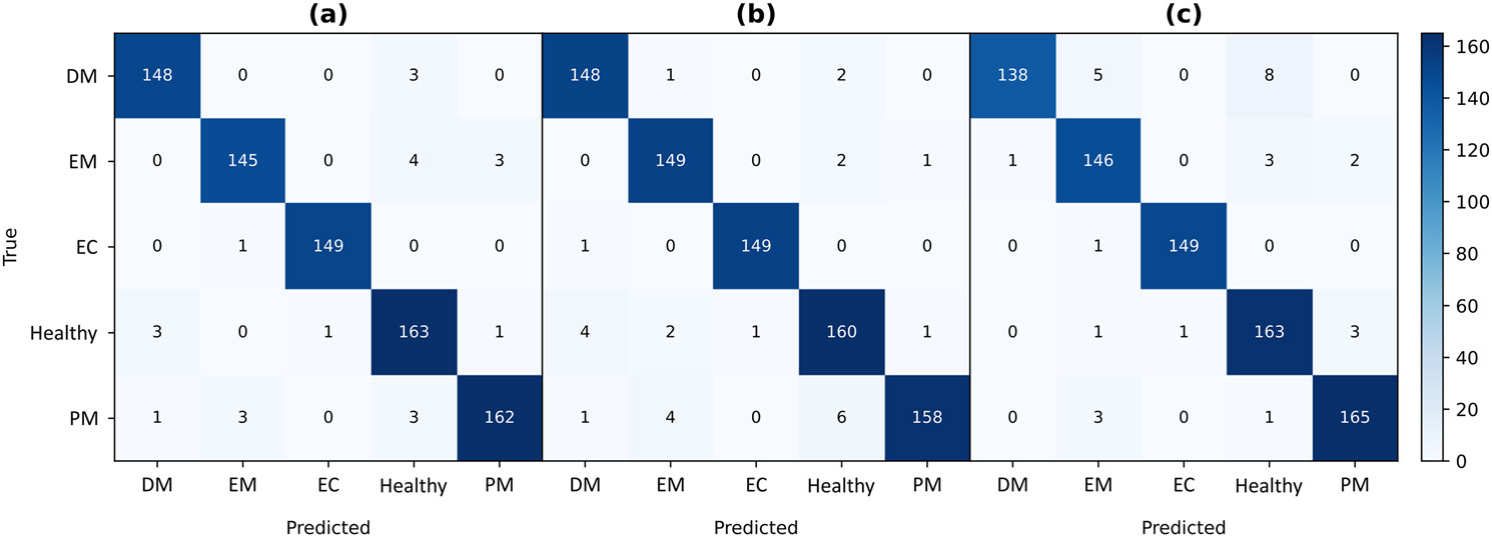
Confusion matrices for the evaluated models: (a) ConvNeXtV2, (b) Swin-T, and (c) ViT-S. DM: Downy Mildew; EM: Erineum Mite; EC: Esca Complex; PM: Powdery Mildew.

The performance in the different classes was high, with F1-scores above 0.94 for all categories and above 0.99 for Esca Complex in all models. The confusion matrices presented in Fig. 4 provide a visual summary of the classification performance for each model. All three architectures show a clear diagonal dominance, indicating that most samples were correctly classified and confirming that all models achieve near-perfect classification, with ConvNeXtV2 showing the cleanest separability across the five grapevine health classes.

**Fig. 5:**
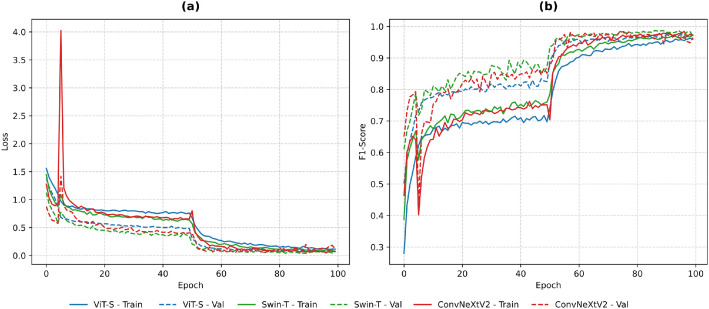
Training and validation loss (a) and F1-score (b) across epochs for ViT-S, Swin-T, and ConvNeXtV2.

The evolution of training and validation loss and F1-score along the training epochs is shown in Fig. 5. All architectures show a rapid decrease in both training and validation loss during the initial epochs, followed by a rise in F1-score, indicating a convergence and the absence of optimization instabilities. The smooth loss trajectories and early stabilization observed in all architectures confirms that the dataset provides visual patterns and well-structured label distributions, enabling efficient learning without overfitting.

**Fig. 6:**
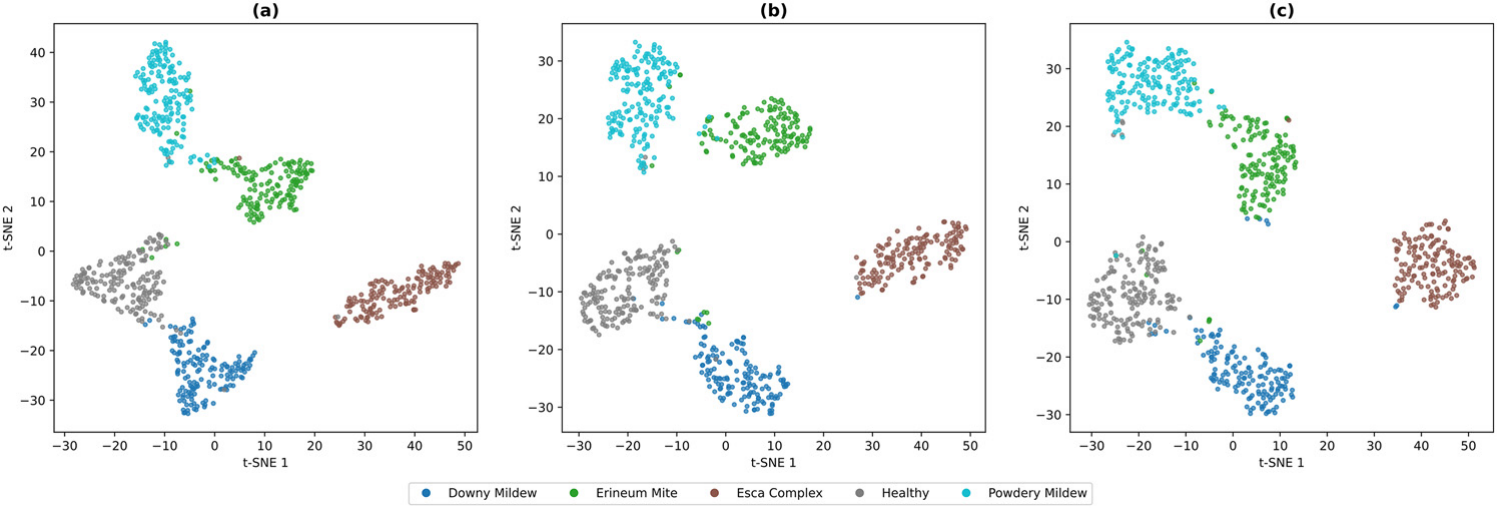
t-SNE visualization of feature embeddings extracted from the three models: (a) ConvNeXtV2, (b) Swin-T, and (c) ViT-S.

To further assess the representational quality of the dataset and the learned feature spaces, a t-SNE visualization [21] of the final embeddings produced by the three models is presented in Fig. 6. The projections reveal well-defined and compact clusters for each disease category, with a minimal overlap between classes, indicating that the dataset provides visually distinctive signs that can be captured by the different architectures. The cluster separation demonstrates that the dataset contains visually discriminative patterns that can be effectively captured by different types of modern deep learning models and the intra-class consistency and inter-class diversity are sufficient to support discriminative feature learning.

Given the stable convergence patterns and high performance achieved in the model architectures, the dataset demonstrates a strong potential for practical applications. Its balanced representation of grapevine disease classes and the robustness of learned features suggest that models trained on this dataset can generalize to real-world vineyard conditions. This makes it suitable not only for benchmarking research in plant pathology and computer vision but also for developing diagnostic tools, such as mobile or embedded systems for automated disease monitoring. The dataset’s quality, variability, and strong class boundaries ensure that models trained on it can provide reliable predictions in field scenarios, supporting decision-making processes in precision viticulture and sustainable crop management.

## Limitations

The dataset consists of RGB images captured under natural lighting conditions, which introduces variation in brightness, shadows, and background elements. All images were acquired manually in open-field environments, without artificial lighting or controlled backgrounds. In most cases, the photographed leaves were fully visible, this approach improves the visibility of symptoms but reduces scene complexity when compared with real-time field conditions, where symptomatic leaves are frequently partially hidden within the canopy. Users who wish to introduce occlusions can create cropped subsets or synthetic occlusions using the original 3000 × 3000 pixel images.

Only three grapevine cultivars (Loureiro, Viosinho and Malvasia Fina) are represented, which restricts coverage of varietal coverage. Several additional white varieties are present in mixed plots, but their contribution is not balanced. The temporal and spatial distribution of diseases is also uneven. Some disease classes are associated with specific vineyards or acquisition dates, as shown in Table 2, Table 3, which restricts the representation of certain combinations of cultivar, location and time.

Disease annotation was based only on visible symptoms, without laboratory confirmation. This may affect diagnostic precision in cases where symptoms overlap or where abiotic stress is present. Some leaves present more than one symptom simultaneously, such as minor erineum mite occurrences on leaves primarily affected by a target disease, which can introduce ambiguity. In addition, some leaves may have been exposed to phytosanitary treatments, which can modify the appearance of symptoms. Information such as leaf age, within‑canopy position and environmental conditions at the time of capture was not recorded. The dataset does not include temporal sequences or symptom progression, since each image represents single time point. The data is prepared for image classification tasks and do not include segmentation masks for symptomatic regions. It also excludes other grapevine diseases such as black rot, botrytis, grapevine leafroll disease, or *flavescence dorée*. These limitations can be mitigated by combining this dataset with existing datasets, including those designed for multi-species disease detection.

The filenames of symptomatic leaves do not contain cultivar names, because some images were acquired in mixed‑variety plots where cultivar identification at the individual leaf level was not reliable. Cultivar information is therefore provided at plot level in Table 3 and in the repository documentation, rather than per image. This reduces the degree of detail available for experimental designs that require per‑leaf cultivar labels. The uneven distribution of diseases by vineyard and acquisition date prevents a temporal or spatial evaluation.

## Ethics Statement

The authors have read and follow the ethical requirements for publication in Data in Brief and confirming that the current work does not involve human subjects, animal experiments, or any data collected from social media platforms.

## CRediT Author Statement

**Fernando Portela:** Conceptualization, Methodology, Formal analysis, Visualization, Writing - Original draft preparation, Formal analysis, Investigation, Data curation. **Gabriel Carneiro**: Formal analysis, Visualization, Data curation, Writing - Review & Editing. **Leilson Ferreira:** Conceptualization, Methodology, Validation, Writing-Reviewing and Editing. **Claudio A. Paredes:** Resources, Writing-Reviewing and Editing, Supervision. **Joaquim J. Sousa:** Resources, Writing-Reviewing and Editing, Supervision, Funding acquisition. **Raul Morais:** Resources, Project administration, Funding acquisition. **Emanuel Peres:** Resources, Project administration, Funding acquisition. **Luís Pádua:** Conceptualization, Methodology, Validation, Investigation, Writing - Reviewing and Editing, Supervision.

## Data Availability

(Original data) (Zenodo).Grapevine Leaves RGB Images of Disease Symptons (Esca, Downy Mildew, Powdery Mildew, and Erineum Mite)
